# Spaces of Ideal Convergent Sequences

**DOI:** 10.1155/2014/134534

**Published:** 2014-01-28

**Authors:** M. Mursaleen, Sunil K. Sharma

**Affiliations:** ^1^Department of Mathematics, Aligarh Muslim University, Aligarh 202002, India; ^2^Department of Mathematics, Model Institute of Engineering & Technology, Kot Bhalwal, J&K 181122, India

## Abstract

In the present paper, we introduce some sequence spaces using ideal convergence and Musielak-Orlicz function **ℳ** = (*M*_*k*_). We also examine some topological properties of the resulting sequence spaces.

## 1. Introduction and Preliminaries 

The notion of ideal convergence was first introduced by Kostyrko et al. [1] as a generalization of statistical convergence which was further studied in topological spaces by Das et al. see [2]. More applications of ideals can be seen in [2, 3]. We continue in this direction and introduce *I*-convergence of generalized sequences with respect to Musielak-Orlicz function.

A family *ℐ* ⊂ 2^*X*^ of subsets of a nonempty set *X* is said to be an ideal in *X* if

1. *ϕ* ∈ *ℐ*,
2. *A*, *B* ∈ *ℐ* imply *A* ∪ *B* ∈ *ℐ*,
3. *A* ∈ *ℐ*, *B* ⊂ *A* imply *B* ∈ *ℐ*,

while an admissible ideal *ℐ* of *X* further satisfies {*x*} ∈ *ℐ* for each *x* ∈ *X*; see [1]. A sequence (*x*_*n*_)_*n*∈*ℕ*_ in *X* is said to be *I*-convergent to *x* ∈ *X*. If for each *ϵ* > 0, the set *A*(*ϵ*) = {*n* ∈ *ℕ* : ||*x*_*n*_ − *x*||≥*ϵ*} belongs to *ℐ*; see [1]. For more details about ideal convergent sequence spaces, see [4–10] and references therein.

Mursaleen and Noman [11] introduced the notion of *λ*-convergent and *λ*-bounded sequences as follows.

Let *λ* = (*λ*_*k*_)_*k*=1_^*∞*^ be a strictly increasing sequence of positive real numbers tending to infinity; that is,

(1) $$\begin{array}{c} 0<\lambda_{0}<\lambda_{1}<\cdots ,\;\;\lambda_{k}⟶\infty \;\text{as}\;\;k⟶\infty . \end{array}$$

The sequence *x* = (*x*_*k*_) ∈ *w* is *λ*-convergent to the number *L*, called the *λ*-limit of *x*, if Λ_*m*_(*x*) → *L*, as *m* → *∞*, where

(2) $$\begin{array}{c} \Lambda_{m}\left(x\right)=\frac{1}{\lambda_{m}}\overset{m}{\underset{k=1}{\sum }}\left(\lambda_{k}-\lambda_{k-1}\right)x_{k}. \end{array}$$

The sequence *x* = (*x*_*k*_) ∈ *w* is *λ*-bounded if sup⁡_*m*_ | Λ_*m*_(*x*)|<*∞*. It is well known [11] that if lim⁡_*m*_ *x*_*m*_ = *a* in the ordinary sense of convergence, then

(3) $$\begin{array}{c} \underset{m}{\lim}\left(\frac{1}{\lambda_{m}}\left(\overset{m}{\underset{k=1}{\sum }}\left(\lambda_{k}-\lambda_{k-1}\right)\left|x_{k}-a\right|\right)\right)=0. \end{array}$$

This implies that

(4) $$\begin{array}{c} \underset{m}{\lim}\left|\Lambda_{m}\left(x\right)-a\right|=\underset{m}{\lim}\left|\frac{1}{\lambda_{m}}\overset{m}{\underset{k=1}{\sum }}\left(\lambda_{k}-\lambda_{k-1}\right)\left(x_{k}-a\right)\right|=0, \end{array}$$

which yields that lim⁡_*m*_Λ_*m*_(*x*) = *a* and hence *x* = (*x*_*k*_) ∈ *w* is *λ*-convergent to *a*.

Let *X* be a linear metric space. A function *p* : *X* → ℝ is called paranorm if

1. *p*(*x*) ≥ 0, for all *x* ∈ *X*,
2. *p*(−*x*) = *p*(*x*), for all *x* ∈ *X*,
3. *p*(*x* + *y*) ≤ *p*(*x*) + *p*(*y*), for all *x*, *y* ∈ *X*,
4. if (*λ*_*n*_) is a sequence of scalars with *λ*_*n*_ → *λ* as *n* → *∞* and (*x*_*n*_) is a sequence of vectors with *p*(*x*_*n*_ − *x*) → 0 as *n* → *∞*, then *p*(*λ*_*n*_ *x*_*n*_ − *λx*) → 0 as *n* → *∞*.

A paranorm *p* for which *p*(*x*) = 0 implies that *x* = 0 is called total paranorm and the pair (*X*, *p*) is called a total paranormed space. It is well known that the metric of any linear metric space is given by some total paranorm (see [12, Theorem 10.4.2, P-183]). For more details about sequence spaces, see [13–15] and references therein.

An Orlicz function *M* is a function which is continuous, nondecreasing, and convex with *M*(0) = 0, *M*(*x*) > 0 for *x* > 0 and *M*(*x*) → *∞* as *x* → *∞*.

Lindenstrauss and Tzafriri [16] used the idea of Orlicz function to define the following sequence space. Let *w* be the space of all real or complex sequences *x* = (*x*_*k*_). Then,

(5) $$\begin{array}{c} \ell_{M}=\left\{x\in w:\overset{\infty }{\underset{k=1}{\sum }}M\left(\frac{\left|x_{k}\right|}{\rho }\right)<\infty \right\} \end{array}$$

which is called an Orlicz sequence space. The space *ℓ*_*M*_ is a Banach space with the norm

(6) $$\begin{array}{c} ||x||=\inf\left\{\rho >0:\overset{\infty }{\underset{k=1}{\sum }}M\left(\frac{\left|x_{k}\right|}{\rho }\right)\leq 1\right\}. \end{array}$$

It is shown in [16] that every Orlicz sequence space *ℓ*_*M*_ contains a subspace isomorphic to *ℓ*_*p*_ (*p* ≥ 1). The Δ_2_-condition is equivalent to *M*(*Lx*) ≤ *kLM*(*x*) for all values of *x* ≥ 0 and for *L* > 1.

A sequence *ℳ* = (*M*_*k*_) of Orlicz function is called a Musielak-Orlicz function see; [17, 18]. A sequence *𝒩* = (*N*_*k*_) defined by

(7) $$\begin{array}{c} N_{k}\left(v\right)=\sup\left\{\left|v\right|u-\left(M_{k}\right):u\geq 0\right\},\;k=1,2,\ldots , \end{array}$$

is called the complementary function of a Musielak-Orlicz function *ℳ*. For a given Musielak-Orlicz function *ℳ*, the Musielak-Orlicz sequence space *t*_*ℳ*_ and its subspace *h*_*ℳ*_ are defined as follows:

(8) $$\begin{array}{c} t_{ℳ}=\left\{x\in w:I_{ℳ}\left(cx\right)<\infty \;\;\text{for}\;\;\text{some}\;\;c>0\right\},\\ h_{ℳ}=\left\{x\in w:I_{ℳ}\left(cx\right)<\infty \;\;\text{for}\;\;\text{all}\;\;c>0\right\}, \end{array}$$

where *I*_*ℳ*_ is a convex modular defined by

(9) $$\begin{array}{c} I_{ℳ}\left(x\right)=\overset{\infty }{\underset{k=1}{\sum }}M_{k}\left(x_{k}\right),\;x=\left(x_{k}\right)\in t_{ℳ}. \end{array}$$

We consider *t*_*ℳ*_ equipped with the Luxemburg norm

(10) $$\begin{array}{c} ||x||=\inf\left\{k>0:I_{ℳ}\left(\frac{x}{k}\right)\leq 1\right\}, \end{array}$$

or equipped with the Orlicz norm

(11) $$\begin{array}{c} {||x||}^{0}=\inf\left\{\frac{1}{k}\left(1+I_{ℳ}\left(kx\right)\right):k>0\right\}. \end{array}$$

Let *ℳ* = (*M*_*k*_) be a Musielak-Orlicz function and let *p* = (*p*_*k*_) be a bounded sequence of positive real numbers. We define the following sequence spaces:

(12) cI(ℳ,Λ,p) ={x=(xk)∈w:I−lim⁡k Mk(|Λk(x)−L|ρ)pk=0,for  some  L  and  ρ>0},c0I(ℳ,Λ,p) ={x=(xk)∈w:I−lim⁡k Mk(|Λk(x)|ρ)pk=0,  for  some  ρ>0},l∞(ℳ,Λ,p)={x=(xk)∈w:sup⁡k Mk(|Λk(x)|ρ)pk<∞,for  some  ρ>0}.

We can write

(13) mI(ℳ,Λ,p)=cI(ℳ,Λ,p)∩l∞(ℳ,Λ,p),m0I(ℳ,Λ,p)=c0I(ℳ,Λ,p)∩l∞(ℳ,Λ,p).

If we take *p* = (*p*_*k*_) = 1, for all *k* ∈ *ℕ*, we have

(14) cI(ℳ,Λ) ={x=(xk)∈w:I−lim⁡k Mk(|Λk(x)−L|ρ)=0,for  some  L  and  ρ>0},c0I(ℳ,Λ)={x=(xk)∈w:I−lim⁡k Mk(|Λk(x)|ρ)=0,for  some  ρ>0},l∞(ℳ,Λ)={x=(xk)∈w:sup⁡k Mk(|Λk(x)|ρ)<∞,for  some  ρ>0}.

The following inequality will be used throughout the paper. If 0 ≤ *p*_*k*_ ≤ sup⁡*p*_*k*_ = *H*, *D* = max⁡(1, 2^*H*−1^), then

(15) $$\begin{array}{c} {\left|a_{k}+b_{k}\right|}^{p_{k}}\leq D\left\{{\left|a_{k}\right|}^{p_{k}}+{\left|B_{k}\right|}^{p_{k}}\right\}, \end{array}$$

for all *k*, and *a*_*k*_, *b*_*k*_ ∈ *ℂ*. Also |*a*|^*p*_*k*_^ ≤ max⁡(1, |*a*|^*H*^) for all *a* ∈ *ℂ*.

The main aim of this paper is to study some ideal convergent sequence spaces defined by a Musielak-Orlicz function *ℳ* = (*M*_*k*_). We also make an effort to study some topological properties and prove some inclusion relations between these spaces.

## 2. Main Results

**Theorem 1**

Let *ℳ* = (*M*_*k*_) be a Musielak-Orlicz function and let *p* = (*p*_*k*_) be a bounded sequence of positive real numbers. Then, the spaces *c* ^*I*^(*ℳ*, Λ, *p*), *c*_0_^*I*^(*ℳ*, Λ, *p*), *m* ^*I*^(*ℳ*, Λ, *p*), and *m*_0_^*I*^(*ℳ*, Λ, *p*) are linear.

**Proof**

Let *x*, *y* ∈ *c* ^*I*^(*ℳ*, Λ, *p*) and let *α*, *β* be scalars. Then, there exist positive numbers *ρ*_1_ and *ρ*_2_ such that

(16) I−lim⁡k Mk(|Λk(x)−L1|ρ1)pk=0, for  some L1∈ℂ,I−lim⁡k Mk(|Λk(y)−L2|ρ2)pk=0, for  some  L2∈ℂ.

For a given *ϵ* > 0, we have

(17) $$\begin{array}{c} D_{1}=\left\{k\in \mathbb{N}:M_{k}{\left(\frac{\left|\Lambda_{k}\left(x\right)-L_{1}\right|}{\rho_{1}}\right)}^{p_{k}}\right\},\\ D_{2}=\left\{k\in \mathbb{N}:M_{k}{\left(\frac{\left|\Lambda_{k}\left(y\right)-L_{2}\right|}{\rho_{2}}\right)}^{p_{k}}\right\}. \end{array}$$

Let *ρ*_3_ = max⁡{2 | *α* | *ρ*_1_, 2 | *β* | *ρ*_2_}. Since *ℳ* = (*M*_*k*_) is nondecreasing convex function, so by using inequality (15), we have

(18) lim⁡k Mk(|Λk((αx+βy)−(αL1+βL2))|ρ3)pk ≤lim⁡k Mk(|α||Λk(x)−L1|ρ3+|β||Λk(y)−L2|ρ3)pk ≤lim⁡k Mk(|Λk(x)−L1|ρ1)pk  +lim⁡k Mk(|Λk(y)−L2|ρ2)pk.

Now, by (17), we have

(19) {k∈ℕ:lim⁡k Mk(|Λk((αx+βy)−(αL1+βL2))|ρ3)pk>ϵ}⊂D1∪D2.

Therefore, *αx* + *βy* ∈ *c* ^*I*^(*ℳ*, Λ, *p*). Hence *c* ^*I*^(*ℳ*, Λ, *p*) is a linear space. Similarly, we can prove that *c*_0_^*I*^(*ℳ*, Λ, *p*), *m* ^*I*^(*ℳ*, Λ, *p*), and *m*_0_^*I*^(*ℳ*, Λ, *p*) are linear spaces.

**Theorem 2**

Let *ℳ* = (*M*_*k*_) be a Musielak-Orlicz function. Then,

(20) $$\begin{array}{c} c_{0}^{I}\left(ℳ,\Lambda ,p\right)\subset c^{I}\left(ℳ,\Lambda ,p\right)\subset l_{\infty }\left(ℳ,\Lambda ,p\right). \end{array}$$

**Proof**

Let *x* ∈ *c* ^*I*^(*ℳ*, Λ, *p*). Then, there exist *L* ∈ *ℂ* and *ρ* > 0 such that

(21) $$\begin{array}{c} I-\underset{k}{\lim}\;M_{k}{\left(\frac{\left|\Lambda_{k}\left(x\right)-L\right|}{\rho }\right)}^{p_{k}}=0. \end{array}$$

We have

(22) $$\begin{array}{c} M_{k}{\left(\frac{\left|\Lambda_{k}\left(x\right)\right|}{2\rho }\right)}^{p_{k}}\leq \frac{1}{2}M_{k}{\left(\frac{\left|\Lambda_{k}\left(x\right)-L\right|}{\rho }\right)}^{p_{k}}+M_{k}\frac{1}{2}{\left(\frac{\left|L\right|}{\rho }\right)}^{p_{k}}. \end{array}$$

Taking supremum over *k* on both sides, we get *x* ∈ *l*_*∞*_(*ℳ*, Λ, *p*). The inclusion *c*_0_^*I*^(*ℳ*, Λ, *p*) ⊂ *c* ^*I*^(*ℳ*, Λ, *p*) is obvious. Thus,

(23) $$\begin{array}{c} c_{0}^{I}\left(ℳ,\Lambda ,p\right)\subset c^{I}\left(ℳ,\Lambda ,p\right)\subset l_{\infty }\left(ℳ,\Lambda ,p\right). \end{array}$$

This completes the proof of the theorem.

**Theorem 3**

Let *ℳ* = (*M*_*k*_) be a Musielak-Orlicz function and let *p* = (*p*_*k*_) be a bounded sequence of positive real numbers. Then, *l*_*∞*_(*ℳ*, Λ, *p*) is a paranormed space with paranorm defined by

(24) $$\begin{array}{c} g\left(x\right)=\inf\left\{\rho >0:\underset{k}{\sup}\;M_{k}{\left(\frac{\left|\Lambda_{k}\left(x\right)\right|}{\rho }\right)}^{p_{k}}\leq 1\right\}. \end{array}$$

**Proof**

It is clear that *g*(*x*) = *g*(−*x*). Since *M*_*k*_(0) = 0, we get *g*(0) = 0. Let us take *x*, *y* ∈ *l*_*∞*_ (*ℳ*, Λ, *p*). Let

(25) B(x)={ρ>0:sup⁡k Mk(|Λk(x)|ρ)pk≤1},B(y)={ρ>0:sup⁡k Mk(|Λk(y)|ρ)pk≤1}.

Let *ρ*_1_ ∈ *B*(*x*) and *ρ*_2_ ∈ *B*(*y*). If *ρ* = *ρ*_1_ + *ρ*_2_, then we have

(26) sup⁡k Mk(|Λk(x+y)|ρ) ≤(ρ1ρ1+ρ2)sup⁡k Mk(|Λk(x)|ρ1)  +(ρ2ρ1+ρ2)sup⁡k Mk(|Λk(y)|ρ2).

Thus, sup⁡_*k*_ *M*_*k*_(|Λ(*x* + *y*)|/(*ρ*_1_ + *ρ*_2_))^*p*_*k*_^ ≤ 1 and

(27) g(x+y)≤inf⁡{(ρ1+ρ2)>0:ρ1∈B(x),ρ2∈B(y)}≤inf⁡⁡{ρ1>0:ρ1∈B(x)} +inf⁡{ρ2>0:ρ2∈B(y)}=g(x)+g(y).

Let *σ* ^*s*^ → *σ*, where *σ*, *σ* ^*s*^ ∈ *ℂ* and let *g*(*x* ^*s*^ − *x*) → 0 as *s* → *∞*. We have to show that *g*(*σ* ^*s*^ *x* ^*s*^ − *σx*) → 0 as *s* → *∞*. Let

(28) B(xs)={ρs>0:sup⁡k Mk(|Λk(xs)|ρs)pk≤1},B(xs−x)={ρs′>0:sup⁡k Mk(|Λk(xs−x)|ρs′)pk≤1}.

If *ρ*_*s*_ ∈ *B*(*x* ^*s*^) and *ρ*_*s*_′ ∈ *B*(*x* ^*s*^ − *x*), then we observe that

(29) Mk(|Λk(σsxs−σx)|ρs|σs−σ|+ρs′|σ|) ≤Mk(|Λk(σsxs−σxs)|ρs|σs−σ|+ρs′|σ|+|(σxs−σx)|ρs|σs−σ|+ρs′|σ|) ≤|σs−σ|ρsρs|σs−σ|+ρs′|σ|Mk((|Λk(xs)|)ρs)  +|σ|ρs′ρs|σs−σ|+ρs′|σ|Mk(|Λk(xs−x)|ρs′).

From the above inequality, it follows that

(30) $$\begin{array}{c} M_{k}{\left(\frac{\left|\Lambda_{k}\left(\sigma^{s}x^{s}-\sigma x\right)\right|}{\rho_{s}\left|\sigma^{s}-\sigma \right|+\rho_{s}^{\prime }\left|\sigma \right|}\right)}^{p_{k}}\leq 1 \end{array}$$

and, consequently,

(31) g(σsxs−σx)≤inf⁡⁡{(ρs|σs−σ|+ρs′|σ|)>0:ρs∈B(xs),ρs′∈B(xs−x)}≤(|σs−σ|)>0inf⁡{ρ>0:ρs∈B(xs)} +(|σ|)>0inf⁡{(ρs′)pn/H:ρs′∈B(xs−x)}⟶0 as  s⟶∞.

This completes the proof.

**Theorem 4**

Let *ℳ*′ = (*M*_*k*_′) and *ℳ*′′ = (*M*_*k*_′′) be Musielak-Orlicz functions that satisfy the Δ_2_-condition. Then,

(i)  *Z*(*ℳ*′′, Λ, *p*)⊆*Z*(*ℳ*′∘*ℳ*′′, Λ, *p*),

(ii)  *Z*(*ℳ*′, Λ, *p*)∩*Z*(*ℳ*′′, Λ, *p*)⊆*Z*(*ℳ*′ + *ℳ*′′, Λ, *p*) for *Z* = *c* ^*I*^, *c*_0_^*I*^, *m* ^*I*^, *m*_0_^*I*^.

**Proof**

(i) Let *x* ∈ *c*_0_^*I*^(*ℳ*′′, Λ, *p*). Then, there exists *ρ* > 0 such that

(32) $$\begin{array}{c} I-\underset{k}{\lim}\;{ℳ}_{k}^{\prime \prime }{\left(\frac{\left|\Lambda_{k}\left(x\right)\right|}{\rho }\right)}^{p_{k}}=0. \end{array}$$

Let *ϵ* > 0 and choose *δ* with 0 < *δ* < 1 such that *M*_*k*_′(*t*) < *ϵ* for 0 ≤ *t* ≤ *δ*. Write *y*_*k*_ = *M*_*k*_′′(|Λ_*k*_(*x*)|/*ρ*)^*p*_*k*_^ and consider

(33) $$\begin{array}{c} \underset{\overset{0\leq y_{k}\leq \delta }{k\in \mathbb{N}}}{\lim}\;M_{k}^{\prime }\left(y_{k}\right)=\underset{\overset{y_{k}\leq \delta }{k\in \mathbb{N}}}{\lim}\;M_{k}^{\prime }\left(y_{k}\right)+\underset{\overset{y_{k}>\delta }{k\in \mathbb{N}}}{\lim}\;M_{k}^{\prime }\left(y_{k}\right). \end{array}$$

Since *ℳ* = (*M*_*k*_) satisfies Δ_2_-condition, we have

(34) $$\begin{array}{c} \underset{\overset{y_{k}\leq \delta }{k\in \mathbb{N}}}{\lim}\;M_{k}^{\prime }\left(y_{k}\right)\leq M_{k}^{\prime }\left(2\right)\underset{\overset{y_{k}\leq \delta }{k\in \mathbb{N}}}{\lim}\left(y_{k}\right). \end{array}$$

For *y*_*k*_ > *δ*, we have

(35) $$\begin{array}{c} y_{k}<\frac{y_{k}}{\delta }<1+\frac{y_{k}}{\delta }. \end{array}$$

Since *ℳ*′ = (*M*_*k*_′) is nondecreasing and convex, it follows that

(36) $$\begin{array}{c} M_{k}^{\prime }\left(y_{k}\right)<M_{k}^{\prime }\left(1+\frac{y_{k}}{\delta }\right)<\frac{1}{2}M_{k}^{\prime }\left(2\right)+\frac{1}{2}\frac{M_{k}^{\prime }\left(2y_{k}\right)}{\delta }. \end{array}$$

Since *ℳ*′ = (*M*_*k*_′) satisfies Δ_2_-condition, we have

(37) $$\begin{array}{c} M_{k}^{\prime }\left(y_{k}\right)<\frac{1}{2}K\frac{y_{k}}{\delta }M_{k}^{\prime }\left(2\right)+\frac{1}{2}K\frac{y_{k}}{\delta }M_{k}^{\prime }\left(2\right)=K\frac{y_{k}}{\delta }M_{k}^{\prime }\left(2\right). \end{array}$$

Hence,

(38) $$\begin{array}{c} \underset{\overset{y_{k}>\delta }{k\in \mathbb{N}}}{\lim}\;M_{k}^{\prime }\left(y_{k}\right)\leq \max\left(1,K\delta^{-1}M_{k}^{\prime }\left(2\right)\right)\underset{\overset{y_{k}\leq \delta }{k\in \mathbb{N}}}{\lim}\left(y_{k}\right). \end{array}$$

From (32), (34), and (38), we have *x* = (*x*_*k*_) ∈ *c*_0_^*I*^(*ℳ*′∘*ℳ*′′, Λ, *p*). Thus, *c*_0_^*I*^(*ℳ*′′, Λ, *p*)⊆*c*_0_^*I*^(*ℳ*′∘*ℳ*′′, Λ, *p*). Similarly, we can prove the other cases.

(ii) Let *x* ∈ *c*_0_^*I*^(*ℳ*′, Λ, *p*)∩*c*_0_^*I*^(*ℳ*′′, Λ, *p*). Then, there exists *ρ* > 0 such that

(39) I−lim⁡k Mk′(|Λk(x)|ρ)pk=0,I−lim⁡k Mk′′(|Λk(x)|ρ)pk=0.

The rest of the proof follows from the following equality:

(40) lim⁡k∈ℕ(Mk′+Mk′′)(|Λk(x)|ρ)pk =lim⁡k∈ℕ Mk′(|Λk(x)|ρ)pk+lim⁡k∈ℕ Mk′′(|Λk(x)|ρ)pk.

**Corollary 5**

Let *ℳ* = (*M*_*k*_) be a Musielak-Orlicz function which satisfies Δ_2_-condition. Then, *Z*(*p*, Λ)⊆*Z*(*ℳ*, Λ, *p*) holds for *Z* = *c* ^*I*^, *c*_0_^*I*^, *m* ^*I*^, and *m*_0_^*I*^.

**Proof**

The proof follows from Theorem 3 by putting *M*_*k*_′′(*x*) = *x* and *M*_*k*_′(*x*) = *M*_*k*_(*x*)  ∀*x* ∈ [0, *∞*).

**Theorem 6**

The spaces *c*_0_^*I*^(*ℳ*, Λ, *p*) and *m*_0_^*I*^(*ℳ*, Λ, *p*) are solid.

**Proof**

We will prove for the space *c*_0_^*I*^(*ℳ*, Λ, *p*Λ). Let *x* ∈ *c*_0_^*I*^(*ℳ*, Λ, *p*). Then, there exists *ρ* > 0 such that

(41) $$\begin{array}{c} I-\underset{k}{\lim}\;M_{k}{\left(\frac{\left|\Lambda_{k}\left(x\right)\right|}{\rho }\right)}^{p_{k}}=0. \end{array}$$

Let (*α*_*k*_) be a sequence of scalars with |*α*_*k*_ | ≤1  ∀*k* ∈ *ℕ*. Then, the result follows from the following inequality:

(42) $$\begin{array}{c} \underset{k}{\lim}\;M_{k}{\left(\frac{\left|\Lambda_{k}\left(\alpha x\right)\right|}{\rho }\right)}^{p_{k}}\leq \underset{k}{\lim}\;M_{k}{\left(\frac{\left|\Lambda_{k}\left(x\right)\right|}{\rho }\right)}^{p_{k}} \end{array}$$

and this completes the proof. Similarly, we can prove for the space *m*_0_^*I*^(*ℳ*, Λ, *p*).

**Corollary 7**

The spaces *c*_0_^*I*^(*ℳ*, Λ, *p*) and *m*_0_^*I*^(*ℳ*, Λ, *p*) are monotone.

**Proof**

It is easy to prove, so we omit the details.

**Theorem 8**

The spaces *c* ^*I*^(*ℳ*, Λ, *p*) and *c*_0_^*I*^(*ℳ*, Λ, *p*) are sequence algebra.

**Proof**

Let *x*, *y* ∈ *c*_0_^*I*^(*ℳ*, Λ, *p*). Then,

(43) I−lim⁡k Mk(|Λk(x)|ρ1)pk=0, for  some  ρ1>0,I−lim⁡k Mk(|Λk(y)|ρ2)pk=0, for  some  ρ2>0.

Let *ρ* = *ρ*_1_ + *ρ*_2_. Then, we can show that

(44) $$\begin{array}{c} I-\underset{k}{\lim}\;M_{k}{\left(\frac{\left|\Lambda_{k}\left(x\cdot y\right)\right|}{\rho }\right)}^{p_{k}}=0. \end{array}$$

Thus, (*x* · *y*) ∈ *c*_0_^*I*^(*ℳ*, Λ, *p*). Hence, *c*_0_^*I*^(*ℳ*, Λ, *p*) is a sequence algebra. Similarly, we can prove that *c* ^*I*^(*ℳ*, Λ, *p*) is a sequence algebra.
